# Functional implications of K63-linked ubiquitination in the iron deficiency response of *Arabidopsis* roots

**DOI:** 10.3389/fpls.2013.00542

**Published:** 2014-01-02

**Authors:** I-Chun Pan, Wolfgang Schmidt

**Affiliations:** ^1^Institute of Plant and Microbial Biology, Academia SinicaTaipei, Taiwan; ^2^Biotechnology Center, National Chung-Hsing UniversityTaichung, Taiwan; ^3^Genome and Systems Biology Degree Program, College of Life Science, National Taiwan UniversityTaipei, Taiwan

**Keywords:** auxin, DNA repair, endocytosis, iron deficiency, K63-linked polyubiquitylation, root hairs

## Abstract

Iron is an essential micronutrient that plays important roles as a redox cofactor in a variety of processes, many of which are related to DNA metabolism. The E2 ubiquitin conjugase UBC13, the only E2 protein that is capable of catalyzing the formation of non-canonical K63-linked ubiquitin chains, has been associated with the DNA damage tolerance pathway in eukaryotes, critical for maintenance of genome stability and integrity. We previously showed that UBC13 and an interacting E3 ubiquitin ligase, RGLG, affect the differentiation of root epidermal cells in *Arabidopsis*. When grown on iron-free media, *Arabidopsis* plants develops root hairs that are branched at their base, a response that has been interpreted as an adaption to reduced iron availability. Mutations in *UBC13A* abolished the branched root hair phenotype. Unexpectedly, mutations in *RGLG* genes caused constitutive root hair branching. Based on recent results that link endocytotic turnover of plasma membrane-bound PIN transporters to K63-linked ubiquitination, we reinterpreted our results in a context that classifies the root hair phenotype of iron-deficient plants as a consequence of altered auxin distribution. We show here that UBC13A/B and RGLG1/2 are involved in DNA damage repair and hypothesize that UBC13 protein becomes limited under iron-deficient conditions to prioritize DNA metabolism. The data suggest that genes involved in combating detrimental effects on genome stability may represent essential components in the plant’s stress response.

## INTRODUCTION

Iron participates in a variety of vital processes as a redox cofactor and is an indispensable element for virtually all organisms. In plants, suboptimal iron availability results in decreased yield and reduced quality of edible plant parts, causing severe economic losses. When plants are the major source of dietary iron, a low iron concentration may pose severe health problems. Iron deficiency-induced anemia (IDA) is the most widespread nutritional disorder worldwide. In severe cases, IDA can affect infant development and increase the risk of maternal and child mortality. Due to the limited solubility of iron in aerated soils, mechanisms have evolved that aid in mobilizing otherwise sparingly soluble Fe(III)oxihydroxides, which represent the prevailing form of iron in most soils at neutral or basic pH. Induction of proton-translocating P-type ATPases decreases the rhizospheric pH, thereby increasing the activity of Fe^3^^+^ by a factor of 1,000 for each unit the pH decreases (Santi et al., 2005; Santi and Schmidt, 2008, 2009). In addition, secretion of iron binding compounds (IBCs) facilitates the mobilization of iron particularly at high pH (Susín et al., 1993; Jin et al., 2007; Fourcroy et al., 2013; Rodríguez-Celma et al.,2013). A further set of supposedly separately regulated responses comprise morphological changes such as the formation of additional root hairs and invaginations of secondary walls in the rhizodermis/hypodermis, responses that were suggested to improve iron acquisition by increasing the absorptive surface area (Schikora and Schmidt, 2001; Schmidt et al., 2003). In contrast to phosphate deficiency, which triggers a nutrient-specific and conserved set of developmental responses including denser and longer root hairs (Ma et al., 2001; Savage et al., 2013) and a dramatically altered root architecture where primary root growth is attenuated and lateral root formation is stimulated (Ticconi et al., 2009), the morphological responses to iron deficiency are less well studied and appear to be less conserved among species. While some plants such as sunflower and cucumber produce very dense root hairs in response to iron deficiency (Landsberg, 1996; Li and Schmidt, 2010), other species such as *Plantago lanceolata* and tomato show only a moderate increase in root hair frequency (Schmidt and Bartels, 1996; Schikora and Schmidt, 2002). Interestingly, both the induction of cell wall invaginations and the formation of extra root hairs are inducible by application of exogenous auxin, suggesting to us that auxin is involved in the induction of these responses (Landsberg, 1996; Schmidt et al., 2003).

In *Arabidopsis*, the morphological responses to iron deficiency are neither pronounced nor uniformly described. Branching of the hairs was described as a major response and interpreted as an alternative to longer and/or denser root hairs as a strategy to increase the surface area of the roots (Müller and Schmidt, 2004). Instead, formation of shorter and misshapen root hairs was described for iron-deficient *Arabidopsis* plants by Dinneny et al. (2008), indicating that subtle differences in media composition and growth conditions may impact the phenotypic readout.

While the physiological responses to iron deficiency are well explored at the molecular level, not much information is available regarding the mechanisms controlling the morphological alterations typical of iron-deficient plants. So far, the only gene with a putative function in iron deficiency-induced formation of root hairs is the ubiquitin conjugase *UBC13*. UBC13 was identified in the root hair zone of iron-deficient cucumber plants by a proteomic approach and cloned using the CODEHOP strategy (Li and Schmidt, 2010). The sequence of UBC13 is highly conserved among eukaryotes, and its function has been related to the error tolerance branch of DNA repair in yeast and *Arabidopsis* and to the NF-κB signal transduction pathway in mammals (Hofmann and Pickart, 1999; Wen et al., 2006; Wu et al., 2009). In *Arabidopsis*, *UBC13* is encoded by two close sequelogs, *UBC13A* (*UBC35*) and *UBC13B* (*UBC36*). UBC13 is the only known E2 ubiquitin conjugating enzyme that is capable of catalyzing the formation of ubiquitin chains linked to K63, a function that appears to be conserved in eukaryotes (Hofmann and Pickart, 1999). In contrast to the formation canonical of ubiquitin chains linked via K48, which target proteins for degradation via the 26S proteasome, proteins conjugated to K63-linked polyubiquitin chains are involved in signaling and in the coordination of cellular processes such as endocytotic trafficking and DNA repair. UBC13 acts in conjunction with E3 ligases such as the RING domain ligase protein RGLG2 that has been shown to interact with UBC13 in *Arabidopsis* (Yin et al., 2007). Non-canonical ubiquitation through K63-linked ubiquitin chains is required for DNA damage tolerance, a pathway that allows the bypass of lesions in the DNA template during replication. The *AtUBC13* genes complemented the yeast *ubc13* null mutant for sensitivity to DNA damaging agents and for spontaneous mutagenesis, suggesting that in *Arabidopsis* UBC13 proteins are involved in the error-free DNA damage tolerance pathway (Wen et al., 2006). The UBC enzyme variant Mms2 is an E2-like protein that interacts with Ubc13 in eukaryotic cells (ubiquitin conjugating enzyme variant, UEV; Hofmann and Pickart, 1999; VanDemark et al., 2001). The *Arabidopsis* genome harbors four Mms2 homologs, *UEV1A* to *UEV1D*, all of which can form stable complexes with UBC13 from yeast and *Arabidopsis* (Wen et al., 2008). For *AtUEV1D* a function in DNA damage response has been experimentally verified (Wen et al., 2008). We here present evidence that both UBC13 and RGLG1/2 are critical in DNA damage repair and hypothesize that the root hair phenotype of iron-deficient plants is caused by diminished availability of RGLG1/2 protein, probably due to stress-induced movement from the plasma membrane to the nucleus and subsequently changed auxin distribution in epidermal cells. We further speculate that under iron-deficient conditions different E2–E3 complexes are favored in the nucleus to prioritize genome stability.

## THE *Arabidopsis* IRON DEFICIENCY ROOT HAIR PHENOTYPE: A CONSEQUENCE OF CHANGES IN AUXIN DISTRIBUTION?

The branched root hair phenotype of iron-deficient plants differs from that of phosphate-deficient plants, which form longer and denser root hairs resulting from restricted longitudinal elongation of root epidermal cells and additional cell fate assignment by increased expression of the Myb-type transcription factor *ETC1* (Ma et al., 2001; Müller and Schmidt, 2004; Savage et al., 2013). Branched root hairs have also been described for auxin-related mutants such as *axr1*, *aux1,* and *axr2*, and for mutants defective in actin filament organization or vesicle transport such as *scn1* or *tip1* (see Guimil and Dunand, 2007 for an overview). Plants harboring mutations in *UBC13A* do not respond to iron deficiency with the formation of branched root hairs. Moreover, *ubc13a ubc13b* double mutants have shorter root hairs compared with the wild type, a trait that was also observed in some auxin-deficient mutants such as *trh1* (Vicente-Agullo et al., 2004; Li and Schmidt, 2010). Transgenic plants overexpressing *UBC13A* (UBC13A OE) showed a phenotype that was essentially similar to that of the wild type when grown on iron-free media, with no further increase in the number of branched hairs. Iron-sufficient UBC13A OE plants are indistinguishable from Col-0 plants, indicating that an iron deficiency signal is required for the induction of root hair branching. Notably, double mutants defective in the expression of the E3 ligases *RGLG1* and *RGLG2* constitutively displayed the branched root hair phenotype (Li and Schmidt, 2010). This was an unexpected result since disruption of the E2/E3 cascade should result in similar phenotypes, regardless of the site of disruption.

Interestingly, the phenotype of *rglg1 rglg2* mutants could be rescued by omitting phosphate from the growth media (Li and Schmidt, 2010). Such a rescue by phosphate deficiency was described for the short root hair phenotype of the auxin transport mutant *trh1* (Müller and Schmidt, 2004). TRH1 is a member of the AtKT/AtKUP/AtHAK family of potassium carriers required for the correct distribution of auxin (Vicente-Agullo et al., 2004). It seems reasonable to speculate that altered auxin metabolism is the cause of the variable phenotype of the mutants grown under the different conditions. In support of this assumption, DR5-GUS reporter lines showed reduced GUS expression under iron-deficient conditions (Lan et al., 2012), indicative of decreased levels of or a diminished responsiveness to auxin. In phosphate-deficient DR5-GUS plants, staining was more intense than under control conditions, indicating increased auxin levels in this growth type. Together these results suggest that compromised auxin transport or metabolism could be the cause for the branched root hair phenotype under iron-deficient conditions.

## A MODEL FOR THE FUNCTION OF K63-LINKED UBIQUITINATION IN ROOT HAIR CELLS

**FIGURE 1:**
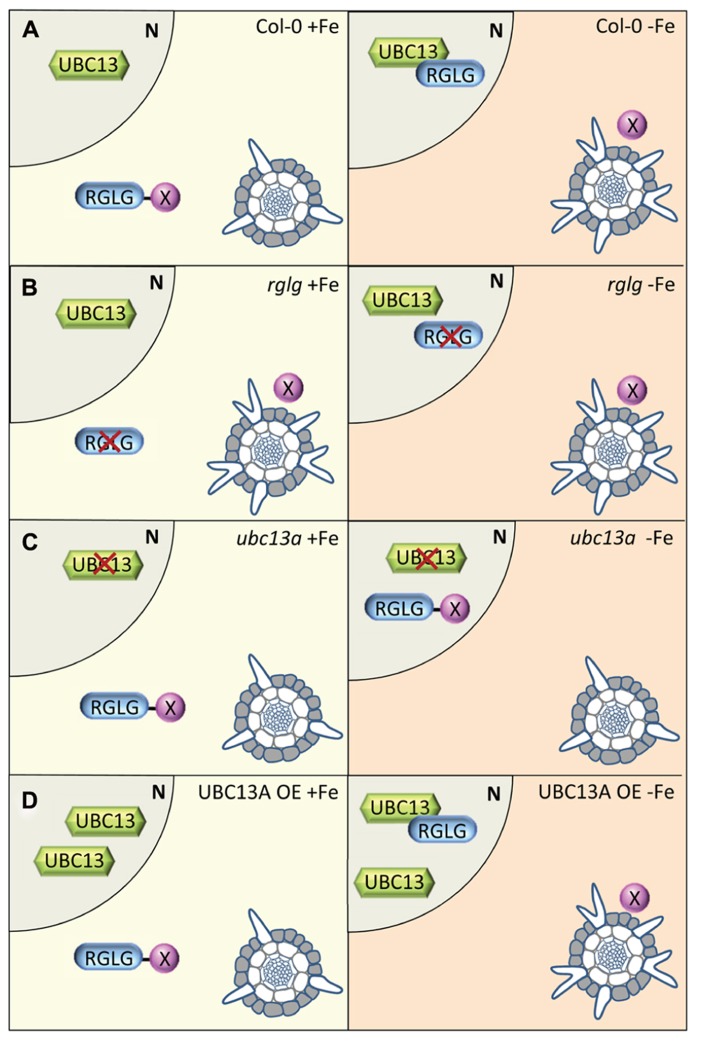
**Model depicting putative interactions of UBC13 and RGLG proteins and their effects on root hair differentiation. (A)** In wild-type roots, protein X is bound to RGLG proteins, preventing the formation of branched root hairs. UBC13 is present, but only under iron-deficient conditions UBC13 interacts with RGLG proteins which move to the nucleus. The interaction of UBC13 with RGLG1/2 releases protein X which leads to the formation of branched hairs. **(B)** In *rglg* mutants, protein X is free both in the presence and absence of iron, causing root hairs to branch independent on the iron supply. **(C)** In root hair cells of *ubc13* plants, no interaction between UBC13 and RGLG1/2 can occur, leaving protein X inactive under both iron-sufficient and iron-deficient conditions. **(D)** Over-expression of UBC13 increases the level of UBC13 protein but does not affect UBC13-RGLG1/2 interaction, resulting in a phenotype similar to that of the wild type.

The following model would explain the results obtained by us (Li and Schmidt, 2010) and others (**Figure 1**). The model is based on observations made in *Arabidopsis* but may also apply to other species. Under iron-sufficient conditions, RGLG1/2 binds a protein X that, when not captured by RGLG1/2, acts as an inhibitor of proper root hair initiation, probably via a reduction of auxin responsiveness. Protein X may act directly or indirectly on auxin distribution. *AtUBC13* is not induced by iron deficiency (Li and Schmidt, 2010) and not much affected by other stresses^1^ and thus likely to be present in iron-sufficient plants in equal amounts. In iron-deficient roots, RGLG1/2 is located to the nucleus where it interacts with UBC13. The UBC13-RGLG1/2 complex formation releases protein X from RGLG, resulting in a decreased auxin concentration (**Figure 1A**). Stress-induced movement of RGLG2 from the plasma membrane to the nucleus has been shown previously (Cheng et al., 2012). In *rglg1 rglg2* mutants, no UBC13-RGLG1/2 interaction is possible, making protein X available both under control and iron-deficient condition. Although K63-linked ubiquitin chain-supporting enzymes such as UBC13 are present in iron-deficient *rglg1 rglg2* plants, they would not affect the abundance of protein X and cause a constitutive branching root hair phenotype (**Figure 1B**). In *ubc13a* plants, branched root hairs are neither formed under iron-sufficient nor under iron-deficient conditions. Adopting the scenario outlined above, protein X remains tightly bound to RGLG1/2, keeping auxin levels (or responsiveness) up and preventing the formation of branched hairs (**Figure 1C**). Over-abundance of UBC13 in UBC13 OE lines is not affecting root hair branching under control conditions (**Figure 1D**). Under iron-deficient conditions, RGLG1/2 moves to the nucleus, releases protein X resulting in a phenotype that is undistinguishable from the wild type.

**FIGURE 2:**
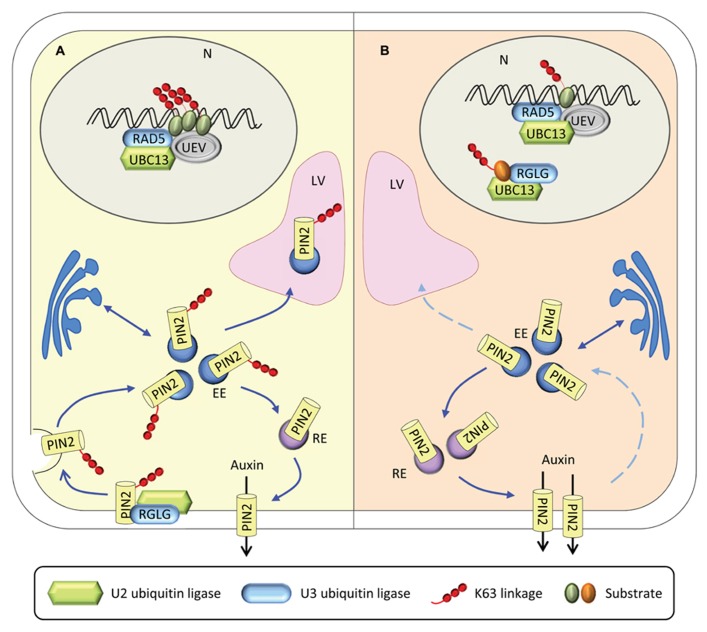
**Experimental evidence for processes associated with the formation of K63-linked ubiquitin chains. (A)** Under control conditions, RGLG mediates the endocytic turnover of PIN2, thereby regulating the distribution of auxin. UBC13-UEV1-RAD5 is involved in housekeeping DNA damage repair. **(B)** Under iron-deficient conditions, RGLG moves to the nucleus where it interacts with UBC13. UBC13-RGLG1/2 might be associated with DNA repair or with other processes related to genome stability. Loss of PIN2 ubiquitination cause a cessation of endocytotic PIN2 cycling, thereby altering auxin distribution. N, nucleus; LV, lytic vacuole; EE early endosome; RE, recycling endosome.

We can only speculate on the nature of protein X. A possible scenario implies that protein X post-translationally regulates auxin distribution or metabolism. The plasma membrane-localized auxin carrier protein PIN2 is constitutively recycled by endocytosis (Benjamins and Scheres, 2008; Grunewald and Friml, 2010). Recently, RGLG proteins were shown to be involved in the control of the proteolytic turnover of PIN2 via K63-linked ubiquitination (Leitner et al., 2012). Loss of PIN2 ubiquitination interferes with vacuolar targeting, stabilizes PIN2, and alters auxin availability in *Arabidopsis* roots. *rglg1 rglg2* mutants had reduced auxin levels and transgenic *rglg1 rglg2* plants carrying the DR5-reporter construct showed reduced auxin responsiveness (Yin et al., 2007). Translated to the model, PIN2 could represent protein X. A possible scenario is outlined in **Figure 2**. Under iron-sufficient conditions, RGLG proteins mediates K63-linked ubiquitination of PIN2, thereby controlling its proteolytic turnover (**Figure 2A**). Loss of PIN2 polyubiquitination, caused for example by a mutation in *RGLG1/2*, causes an arrest of endocytotic cycling of PIN2, decreased auxin levels and, ultimately, branching of root hairs. Such a scenario may apply to iron-deficient plants in which UBC13 is recruited by RGLG1/2 in the nucleus, compromising the ubiquitination of PIN2, which accumulates in the cell (**Figure 2B**). Recruitment of UBC13 may be facilitated by translocation of RGLG from the plasma membrane to the nucleus.

UBC13 was suggested to be involved in the error-free DNA damage repair pathway (Wen et al., 2006). In yeast, this function is fulfilled by an Ubc13-Mms2-Rad5-mediated polyubiquitination of the homotrimeric protein complex proliferating cell nuclear antigen (PCNA); in humans, PCNA ubiquitination is catalyzed by the Rad5 homologs HLTF and SHPRH (Ulrich, 2007; Chang and Cimprich, 2009). A role for AtRAD5A in DNA repair has been demonstrated (Chen et al., 2008; Mannuss et al., 2010; Wang et al., 2011) and an involvement of UBC13-UEV1 and AtRAD5A in this pathway in *Arabidopsis* represents a likely scenario.

In *ubc13a* mutant plants, several iron-responsive genes are less induced than in the wild type (Li and Schmidt, 2010), indicative of an involvement of UBC13A in processes that utilizes iron. For example, induction of the transcription factor *bHLH38*, an essential regulator of the iron deficiency response, was markedly less pronounced in roots of the *ubc13a* mutant. Notably, under iron-deficient conditions the expression of two genes encoding the iron storage proteins *FER1* and *FER2* was higher in *ubc13a* plants when compared with the wild type, suggesting a higher iron status of the mutant. These results may be interpreted in a sense that more iron is available if the UBC13-RGLG pathway is not engaged. Based on the conserved function of UBC13 in DNA damage repair among eukaryotes, it can be assumed that the UBC13-RGLG1/2 complex participates in DNA damage repair.

**FIGURE 3:**
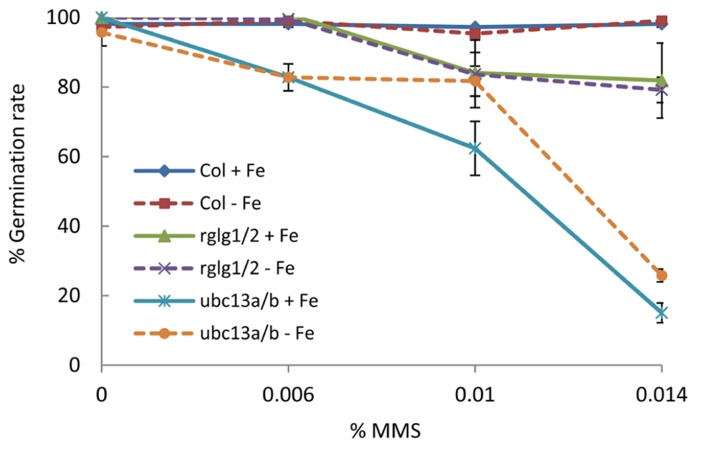
**Analysis of DNA damage response during seed germination.** Seeds of wild-type plants, *rglg1 rglg2*, and *ubc13a ubc13b* double mutants were placed on either iron-replete or iron-deplete media containing 0, 0.006%, 0.01%, and 0.014% MMS. Data points indicate the percentage of seed germination after 5 days with SD (*n* = 36).

To test our hypothesis, we germinated seeds from wild-type plants, *rglg1 rglg2*, and *ubc13a ubc13b* double mutants on media containing various concentrations of the DNA-damaging agent methyl methanesulfonate (MMS). While wild-type plants were largely unaffected both under iron-sufficient and iron-deficient conditions, the germination rate of *ubc13a ubc13b* double mutants showed a dramatic decrease in germination rate which was more pronounced under iron-deficient conditions (**Figure 3**). *rglg1 rglg2* mutants showed a less pronounced and iron-independent decrease in seed germination (**Figure 3**). These data are consistent with the assumption that under iron-deficient conditions RGLG1/2 is translocated into the nucleus where it may associate with UBC13 and other E2 proteins, thereby diminishing the efficiency of UBC13 in DNA damage tolerance. UBC13 may have critical, currently unknown functions in DNA metabolism in conjunction with RGLG1/2, probably associated with genome stability (**Figure 2B**).

## THE ROLE OF IRON IN DNA SYNTHESIS AND REPAIR

Iron is required for several DNA-related processes. For example, ribonucleotide reductases (RNRs) require iron as an essential cofactor and iron deficiency leads to reduced RNR activity (Cavanaugh et al., 1985; Saletta et al., 2011). RNRs activity is linked to DNA synthesis and tightly regulated in response to iron deficiency in yeast and mammals to assure accurate DNA replication (Furukawa et al., 1992). In yeast, a post-transcriptional regulatory mechanism promotes destabilization of mRNAs involved in non-essential pathways and degradation of such transcripts via AU-rich element (ARE)-mediated decay (AMD), and supports DNA synthesis and repair via activation of RNR by the Cth2 protein (Martínez-Pastor et al., 2013). Interestingly, many of the down-regulated transcripts are related to the tricarboxylic acid cycle or participate in mitochondrial electron transport, switching respiration to fermentative metabolism (Sanvisens et al., 2011), a response that has also been described for iron-deficient *Arabidopsis* roots (Thimm et al., 2001). Also, storage of iron in the vacuole via CCC1 is inhibited by this pathway, a further parallel to *Arabidopsis* roots in which CCC1-like vacuolar iron transporters are down-regulated in response to iron deficiency (Gollhofer et al., 2011).

In humans, iron deficiency leads to G1/S cell cycle arrest and to the induction of several members of the growth arrest and DNA damage (GADD) gene family members, probably associated with DNA damage resulting from iron deficiency (Saletta et al., 2011). In addition, DNA replication and repair depends on Fe–S clusters (Netz et al., 2010; Pokharel and Campbell, 2012) the synthesis of which is impaired upon iron deficiency (Chen et al., 2004). Defects in Fe–S cluster biogenesis result in genome instability (Veatch et al., 2009). Not surprisingly, mitochondrial dysfunction associated with compromised biogenesis of Fe–S clusters is tightly linked to iron homeostasis (Veatch et al., 2009).

DNA damage applies to the sites of the storage of genetic material, i.e., the nucleus, plastids, and mitochondria. More recently, nucleoli have been associated with a function of sensing DNA damage and as a storage facility for DNA repair proteins (Antoniali et al., 2013). In plants, iron is concentrated in the nuclei, particularly in nuclear substructures that were identified as the nucleoli by Perls/DAB and DAPI co-staining (Roschzttardtz et al., 2011). The nucleolus was described as a central hub for the coordination of stress responses such as DNA repair (Boulon et al., 2010; Antoniali et al., 2013), and for several DNA-repair-related proteins (Gao et al., 2003; Guo et al., 2008). While in plants such information is not available, we speculate that the high iron concentrations in nuclei/nucleoli are important for prioritizing DNA replication and repair when iron becomes limited.

## CONCLUSION

In summary, the re-interpretation of the data outlined above hints at a new facet of the iron deficiency response of *Arabidopsis*, which might also be important for other plant species and may thus represent a general aspect in the adaptation of plants to low iron availability. Under iron shortage, DNA-related processes such as replication and repair might be prioritized to secure essential housekeeping functions. While alternative scenarios such as a role of UBC13 in root development that is not linked to DNA repair cannot be ruled out at present, the fact that *ubc13a* mutants appear to have a higher, healthier iron status implies that activation of the DNA damage pathway occurs at the expense of other genes encoding iron-containing proteins. This situation is similar to what has been reported for yeast where iron deficiency limits iron utilization in energy-generating pathways via the action of the RNA-binding protein Cth2 to avoid that essential processes such as DNA synthesis and repair and thus genome integrity are compromised (Martínez-Pastor et al., 2013). While the evidence reported here is indirect and awaits experimental validation, it is tempting to assume that, similar to yeast, post-transcriptional mechanisms that bias the translation of messenger RNAs encoding proteins involved in essential iron-requiring processes such as DNA replication and repair also occur in *Arabidopsis* and other plants. Our initial experiments indicate that both UBC13 and RGLG are involved in DNA repair, but only for *ubc13a ubc13b* mutants iron deficiency severed the effect of MMS. This is consistent with a competition for UBC13 protein in the nucleus due to stress-induced translocation of RGLG1/2 to the nucleus. The role of RGLG1/2 under iron-deficient conditions awaits clarification, but from our results a function for RGLG1/2 in genome stability appears to be plausible. Further experiments addressing the fitness of plants treated with DNA-damaging agents and grown with different iron supply may falsify or validate our hypothesis. The effects associated with adverse environmental condition on genome stability are generally understudied and, despite big steps forward in the past few years (reviewed by Waterworth et al., 2011), not fully understood. From the example outlined here for iron deficiency it might be inferred that mechanisms that are involved in combating the constant assault of environmental stresses on genome stability may be an important component of the plant stress response.

## MATERIALS AND METHODS

*Arabidopsis thaliana* (L.) Heynh Columbia (Col-0) ecotype was used as the wild-type. Seeds of the wild type, *ubc13a* and *ubc13b* mutants were obtained from the Arabidopsis Biological Resource Centre (Ohio State University), *ubc13a ubc13b* double mutants were generated by genetic crossing. The *rglg1 rglg2* mutant lines were provided by Dr. Andreas Bachmeir (University of Vienna). Seeds were surface-sterilized and sown on media as described by Estelle and Somerville (1987), supplemented with 0%, 0.006%, 0.01%, and 0.014% MMS either with or without 40 μM FeEDTA, and stored at 4°C for 2 days. A total of 36 seeds were placed on each plate, three plates were used for each treatment. After 5 days, seed germination was determined and normalized with the rate observed on iron-replete standard without MMS.

## Conflict of Interest Statement

The authors declare that the research was conducted in the absence of any commercial or financial relationships that could be construed as a potential conflict of interest.

## Footnotes

1

## References

[B1] Antoniali G. Lirussi L. Poletto M. Tell G. (2013). Emerging roles of the nucleolus in regulating the DNA damage response: the noncanonical DNA repair enzyme APE1/Ref-1 as a paradigmatical example. *Antioxid. Redox Signal.* 10.1089/ars.2013.5491 PMC3901381 23879289

[B2] Benjamins R. Scheres B. (2008). Auxin: the looping star in plant development. *Annu. Rev. Plant Biol.* 59 443–465 10.1146/annurev.arplant.58.032806.103805 18444904

[B3] Boulon S. Westman B. J. Hutten S. Boisvert F. M. Lamond A. I. (2010). The nucleolus under stress. *Mol. Cell* 40 216–227 10.1016/j.molcel.2010.09.024 20965417 PMC2987465

[B4] Cavanaugh P. F. Jr. Porter C. W. Tukalo D. Frankfurt O. S. Pavelic Z. P. Bergeron R. J. (1985). Characterization of L1210 cell growth inhibition by the bacterial iron chelators parabactin and compound II. *Cancer Res.* 45 4754–4759 4027962

[B5] Chang D. J. Cimprich K. A. (2009). DNA damage tolerance: when it’s OK to make mistakes. *Nat. Chem. Biol.* 5 82–90 10.1038/nchembio.139 19148176 PMC2663399

[B6] Chen I. P. Mannuss A. Orel N. Heitzeberg F. Puchta H. (2008). A homolog of ScRAD5 is involved in DNA repair and homologous recombination in *Arabidopsis*. *Plant Physiol.* 146 1786–1796 10.1104/pp.108.116806 18310306 PMC2287350

[B7] Chen O. S. Crisp R. J. Valachovic M. Bard M. Winge D. R. Kaplan J. (2004). Transcription of the yeast iron regulon does not respond directly to iron but rather to iron–sulfur cluster biosynthesis. *J. Biol. Chem.* 279 29513–29518 10.1074/jbc.M403209200 15123701

[B8] Cheng M. C. Hsieh E. J. Chen J. H. Chen H. Y. Lin T. P. (2012). *Arabidopsis* RGLG2, functioning as a RING E3 ligase, interacts with AtERF53 and negatively regulates the plant drought stress response. *Plant Physiol.* 158 363–375 10.1104/pp.111.189738 22095047 PMC3252077

[B9] Dinneny J. R. Long T. A. Wang J. Y. Jung J. W. Mace D. Pointer S. (2008). Cell identity mediates the response of *Arabidopsis* roots to abiotic stress. *Science* 320 942–945 10.1126/science.1153795 18436742

[B10] Estelle M. A. Somerville C. (1987). Auxin-resistant mutants of *Arabidopsis thaliana* with an altered morphology. *Mol. Gen. Genet.* 206 200–206 10.1007/BF00333575

[B11] Fourcroy P. Siso-Terraza P. Sudre D. Saviron M. Reyt G. Gaymard F. (2013). Involvement of the ABCG37 transporter in secretion of scopoletin and derivatives by *Arabidopsis* roots in response to iron deficiency. *New Phytol.* 201 155–167 10.1111/nph.12471 24015802

[B12] Furukawa T. Naitoh Y. Kohno H. Tokunaga R. Taketani S. (1992). Iron deprivation decreases ribonucleotidereductase activity and DNA synthesis. *Life Sci.* 50 2059–2065 10.1016/0024-3205(92)90572-7 1608289

[B13] Gao H. Chen X. B. Mcgowan C. H. (2003). Mus81 endonuclease localizes to nucleoli and to regions of DNA damage in human S-phase cells. *Mol. Biol. Cell* 14 4826–4834 10.1091/mbc.E03-05-0276 14638871 PMC284787

[B14] Gollhofer J. Schlawicke C. Jungnick N. Schmidt W. Buckhout T. J. (2011). Members of a small family of nodulin-like genes are regulated under iron deficiency in roots of *Arabidopsis thaliana*. *Plant Physiol. Biochem.* 49 557–564 10.1016/j.plaphy.2011.02.011 21411332

[B15] Grunewald W. Friml J. (2010). The march of the PINs: developmental plasticity by dynamic polar targeting in plant cells. *EMBO J.* 29 2700–2714 10.1038/emboj.2010.181 20717140 PMC2924653

[B16] Guimil S. Dunand C. (2007). Cell growth and differentiation in *Arabidopsis* epidermal cells. *J. Exp. Bot.* 58 3829–3840 10.1093/jxb/erm253 18162628

[B17] Guo Z. Qian L. Liu R. Dai H. Zhou M. Zheng L. (2008). Nucleolar localization and dynamic roles of flap endonuclease 1 in ribosomal DNA replication and damage repair. *Mol. Cell. Biol.* 28 4310–4319 10.1128/MCB.00200-08 18443037 PMC2447149

[B18] Hofmann R. M. Pickart C. M. (1999). Noncanonical MMS2-encoded ubiquitin-conjugating enzyme functions in assembly of novel polyubiquitin chains for DNA repair. *Cell* 96 645–653 10.1016/S0092-8674(00)80575-9 10089880

[B19] Jin C. W. You G. Y. He Y. F. Tang C. X. Wu P. Zheng S. J. (2007). Iron deficiency-induced secretion of phenolics facilitates the reutilization of root apoplastic iron in red clover. *Plant Physiol.* 144 278–285 10.1104/pp.107.095794 17369430 PMC1913808

[B20] Lan P. Li W. F. Wen T. N. Schmidt W. (2012). Quantitative phosphoproteome profiling of iron-deficient *Arabidopsis* roots. *Plant Physiol.* 159 403–417 10.1104/pp.112.193987 22438062 PMC3375974

[B21] Landsberg E. C. (1996). Hormonal regulation of iron-stress response in sunflower roots: a morphological and cytological investigation. *Protoplasma* 194 69–80 10.1007/BF01273169

[B22] Leitner J. Retzer K. Korbei B. Luschnig C. (2012). Dynamics in PIN2 auxin carrier ubiquitylation in gravity-responding *Arabidopsis* roots. *Plant Signal. Behav.* 7 1271–1273 10.4161/psb.21715 22902683 PMC3493411

[B23] Li W. Schmidt W. (2010). A lysine-63-linked ubiquitin chain-forming conjugase, UBC13, promotes the developmental responses to iron deficiency in *Arabidopsis* roots. *Plant J.* 62 330–343 10.1111/j.1365-313X.2010.04150.x 20113438

[B24] Ma Z. Walk T. C. Marcus A. Lynch J. P. (2001). Morphological synergism in root hair length, density, initiation and geometry for phosphorus acquisition in *Arabidopsis thaliana*: a modeling approach. *Plant Soil* 236 221–235 10.1023/A:1012728819326

[B25] Mannuss A. Dukowic-Schulze S. Suer S. Hartung F. Pacher M. Puchta H. (2010). RAD5A, RECQ4A, and MUS81 have specific functions in homologous recombination and define different pathways of DNA repair in *Arabidopsis thaliana*. *Plant Cell* 22 3318–3330 10.1105/tpc.110.078568 20971895 PMC2990144

[B26] Martínez-Pastor M. T. De Llanos R. Romero A. M. Puig S. (2013). Post-transcriptional regulation of iron homeostasis in *Saccharomyces cerevisiae*. *Int. J. Mol. Sci.* 14 15785–15809 10.3390/ijms140815785 23903042 PMC3759886

[B27] Müller M. Schmidt W. (2004). Environmentally induced plasticity of root hair development in *Arabidopsis*. *Plant Physiol.* 134 409–419 10.1104/pp.103.029066 14730071 PMC371035

[B28] Netz D. J. Stumpfig M. Dore C. Muhlenhoff U. Pierik A. J. Lill R. (2010). Tah18 transfers electrons to Dre2 in cytosolic iron–sulfur protein biogenesis. *Nat. Chem. Biol.* 6 758–765 10.1038/nchembio.432 20802492

[B29] Pokharel S. Campbell J. L. (2012). Cross talk between the nuclease and helicase activities of Dna2: role of an essential iron–sulfur cluster domain. *Nucleic Acids Res.* 40 7821–7830 10.1093/nar/gks534 22684504 PMC3439918

[B30] Rodríguez-Celma J. Lin W. D. Fu G. M. Abadía J. López-Míllan A. F. Schmidt W. (2013). Mutually exclusive alterations in secondary metabolism are critical for the uptake of insoluble iron compounds by *Arabidopsis* and *Medicago truncatula*. *Plant Physiol.* 162 1473–1485 10.1104/pp.113.220426 23735511 PMC3707556

[B31] Roschzttardtz H. Grillet L. Isaure M. P. Conejero G. Ortega R. Curie C. (2011). Plant cell nucleolus as a hot spot for iron. *J. Biol. Chem.* 286 27863–27866 10.1074/jbc.C111.269720 21719700 PMC3151030

[B32] Saletta F. Suryo Rahmanto Y. Siafakas A. R. Richardson D. R. (2011). Cellular iron depletion and the mechanisms involved in the iron-dependent regulation of the growth arrest and DNA damage family of genes. *J. Biol. Chem.* 286 35396–35406 10.1074/jbc.M111.273060 21852233 PMC3195607

[B33] Santi S. Cesco S. Varanini Z. Pinton R. (2005). Two plasma membrane H^(^^+^^)^-ATPase genes are differentially expressed in iron-deficient cucumber plants. *Plant Physiol. Biochem.* 43 287–292 10.1016/j.plaphy.2005.02.007 15854837

[B34] Santi S. Schmidt W. (2008). Laser microdissection-assisted analysis of the functional fate of iron deficiency-induced root hairs in cucumber. *J. Exp. Bot.* 59 697–704 10.1093/jxb/erm351 18316319

[B35] Santi S. Schmidt W. (2009). Dissecting iron deficiency-induced proton extrusion in *Arabidopsis* roots. *New Phytol.* 183 1072–1084 10.1111/j.1469-8137.2009.02908.x 19549134

[B36] Sanvisens N. Bano M. C. Huang M. Puig S. (2011). Regulation of ribonucleotidereductase in response to iron deficiency. *Mol. Cell* 44 759–769 10.1016/j.molcel.2011.09.021 22152479 PMC3240860

[B37] Savage N. Yang T. J. Chen C. Y. Lin K.-L. Monk N. A. M. Schmidt W. (2013). Positional signaling and expression of ENHANCER OF TRY AND CPC1 are tuned to increase root hair density in response to phosphate deficiency in *Arabidopsis thaliana*. *PLoS ONE* 8:e75452 10.1371/journal.pone.0075452 PMC3794009 24130712

[B38] Schikora A. Schmidt W. (2001). Iron stress-induced changes in root epidermal cell fate are regulated independently from physiological responses to low iron availability. *Plant Physiol.* 125 1679–1687 10.1104/pp.125.4.1679 11299349 PMC88825

[B39] Schikora A. Schmidt W. (2002). Formation of transfer cells and H^(^^+^^)^-ATPase expression in tomato roots under P and Fe deficiency. *Planta* 215 304–311 10.1007/s00425-002-0738-0 12029480

[B40] Schmidt W. Bartels M. (1996). Formation of root epidermal transfer cells in Plantago. *Plant Physiol.* 110 217–225 12226179 10.1104/pp.110.1.217 PMC157712

[B41] Schmidt W. Michalke W. Schikora A. (2003). Proton pumping by tomato roots. Effect of Fe deficiency and hormones on the activity and distribution of plasma membrane H^+^-ATPase in rhizodermal cells. *Plant Cell Environ.* 26 361–370 10.1046/j.1365-3040.2003.00967.x

[B42] Susín S. Abian J. Sanchez-Baeza F. Peleato M. L. Abadía A. Gelpi E. (1993). Riboflavin 3′- and 5′sulfate, two novel flavins accumulating in the roots of iron-deficient sugar beet (*Beta vulgaris*). *J. Biol. Chem.* 268 20958–20965 8407931

[B43] Thimm O. Essigmann B. Kloska S. Altmann T. Buckhout T. J. (2001). Response of *Arabidopsis* to iron deficiency stress as revealed by microarray analysis. *Plant Physiol.* 127 1030–1043 10.1104/pp.010191 11706184 PMC129273

[B44] Ticconi C. A. Lucero R. D. Sakhonwasee S. Adamson A. W. Creff A. Nussaume L. (2009). ER-resident proteins PDR2 and LPR1 mediate the developmental response of root meristems to phosphate availability. *Proc. Natl. Acad. Sci. U.S.A.* 106 14174–14179 10.1073/pnas.0901778106 19666499 PMC2723163

[B45] Ulrich H. D. (2007). Conservation of DNA damage tolerance pathways from yeast to humans. *Biochem. Soc. Trans.* 35 1334–1337 10.1042/BST0351334 17956345

[B46] VanDemark A. P. Hofmann R. M. Tsui C. Pickart C. M. Wolberger C. (2001). Molecular insights into polyubiquitin chain assembly: crystal structure of the Mms2/Ubc13 heterodimer. *Cell* 105 711–720 10.1016/S0092-8674(01)00387-7 11440714

[B47] Veatch J. R. McMurray M. A. Nelson Z. W. Gottschling D. E. (2009). Mitochondrial dysfunction leads to nuclear genome instability via an iron–sulfur cluster defect. *Cell* 137 1247–1258 10.1016/j.cell.2009.04.014 19563757 PMC2759275

[B48] Vicente-Agullo F. Rigas S. Desbrosses G. Dolan L. Hatzopoulos P. Grabov A. (2004). Potassium carrier TRH1 is required for auxin transport in *Arabidopsis* roots. *Plant J.* 40 523–535 10.1111/j.1365-313X.2004.02230.x 15500468

[B49] Wang S. Wen R. Shi X. Lambrecht A. Wang H. Xiao W. (2011). RAD5a and REV3 function in two alternative pathways of DNA-damage tolerance in *Arabidopsis*. *DNA Repair* 10 620–628 10.1016/j.dnarep.2011.04.009 21549648

[B50] Waterworth W. M. Drury G. E. Bray C. M. West C. E. (2011). Repairing breaks in the plant genome: the importance of keeping it together. *New Phytol.* 192 805–822 10.1111/j.1469-8137.2011.03926.x 21988671

[B51] Wen R. Newton L. Li G. Y. Wang H. Xiao W. (2006). *Arabidopsis thaliana* UBC13: implication of error-free DNA damage tolerance and Lys63-linked polyubiquitylation in plants. *Plant Mol. Biol.* 61 241–253 10.1007/s11103-006-0007-x 16786304

[B52] Wen R. Torres-Acosta J. A. Pastushok L. Lai X. Pelzer L. Wang H. (2008). *Arabidopsis* UEV1D promotes lysine-63-linked polyubiquitination and is involved in DNA damage response. *Plant Cell* 20 213–227 10.1105/tpc.107.051862 18178771 PMC2254933

[B53] Wu X. F. Yamamoto M. Akira S. Sun S. C. (2009). Regulation of hematopoiesis by the K63-specific ubiquitin-conjugating enzyme Ubc13. *Proc. Natl. Acad. Sci. U.S.A.* 106 20836–20841 10.1073/pnas.0906547106 19926860 PMC2791607

[B54] Yin X. J. Volk S. Ljung K. Mehlmer N. Dolezal K. Ditengou F. (2007). Ubiquitin lysine 63 chain forming ligases regulate apical dominance in *Arabidopsis*. *Plant Cell* 19 1898–1911 10.1105/tpc.107.052035 17586653 PMC1955712

